# Supplementary material for: G6PD and ACSL3 are synthetic lethal partners of NF2 in Schwann cells
Source: Nat Commun. 2024 Jun 15;15:5115. doi: 10.1038/s41467-024-49298-7 (PMC11180199; doi:10.1038/s41467-024-49298-7)
Supplement: Supplementary file 1 — Supplementary Information [file 41467_2024_49298_MOESM1_ESM.pdf]

Supplementary Information

G6PD and ACSL3 are synthetic lethal partners of NF2 in Schwann cells

Athena Kyrkou, Robert Valla et al.

Contents

Supplementary Figures .... p. 2

Uncropped Immunoblots of Supplementary Figures .... p. 19

Supplementary Information References... p. 34

Supplementary Figure 1

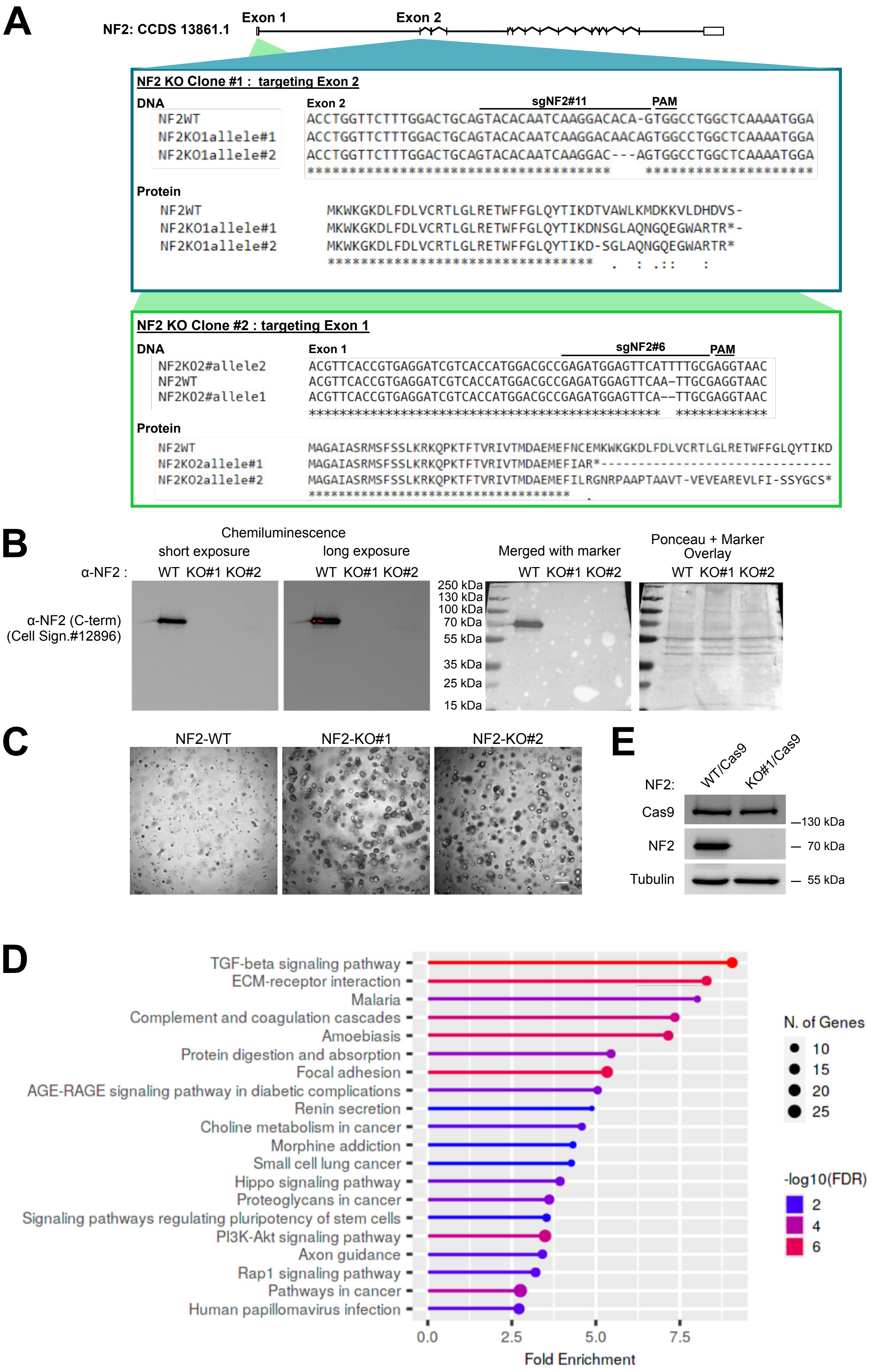

Supplementary Figure 1: Characterization of NF2 KO Schwann cell lines.

(A) Molecular characterization of the two NF2 KO Schwann cell lines (DNA and resulting protein truncations) targeting exons 1 and 2.

(B) NF2-KO cells do not express a truncated form of NF2. Immunoblot of NF2-WT and NF2-KO cells with an antibody that detects the c-terminal region of NF2.

(C) NF2-KO Schwann cells, but not the NF2-WT parental line, can form anchorage-independent clones in soft agar (7 days).

(D) Gene Ontology enrichment on genes differentially expressed in NF2-KO#1 cells compared to NF2-WT cells ($|log2FC|>2$ and Benjamini-Hochberg $p < 0.01$), analysed with ShinyGo [1].

(E) Immunoblot analysis of Cas9 expression in the cell lines that were used for the synthetic lethality screen.

Source data are provided as a Source Data file.

Supplementary Figure 2

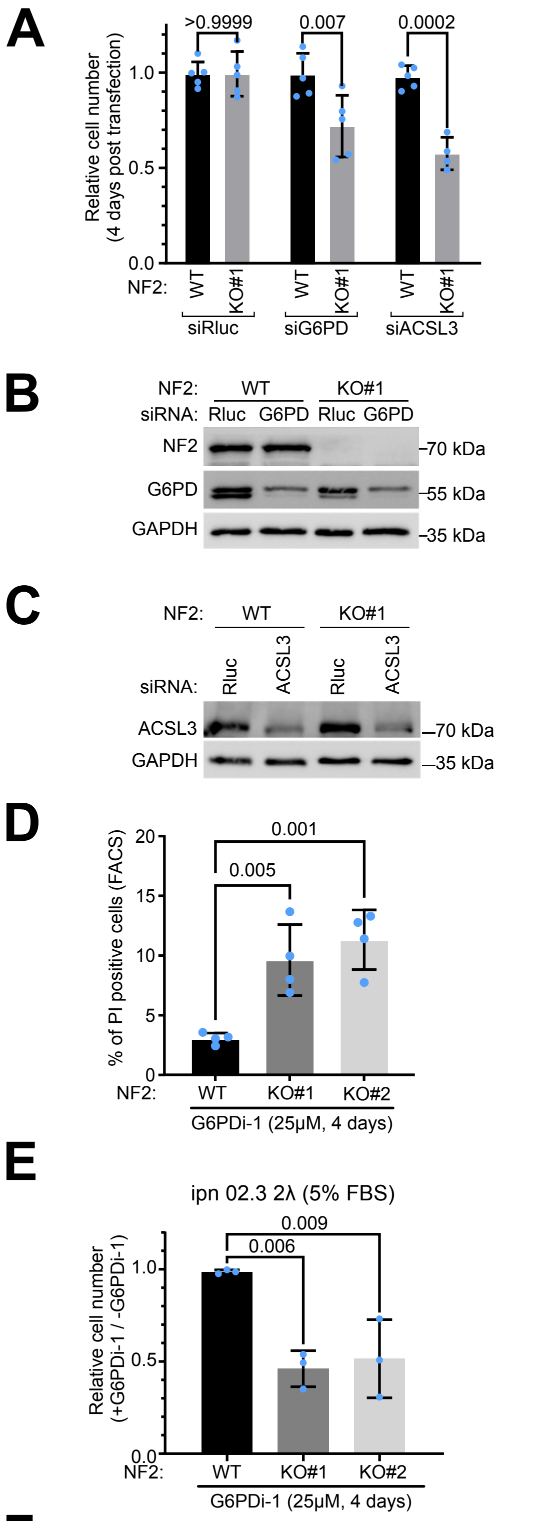

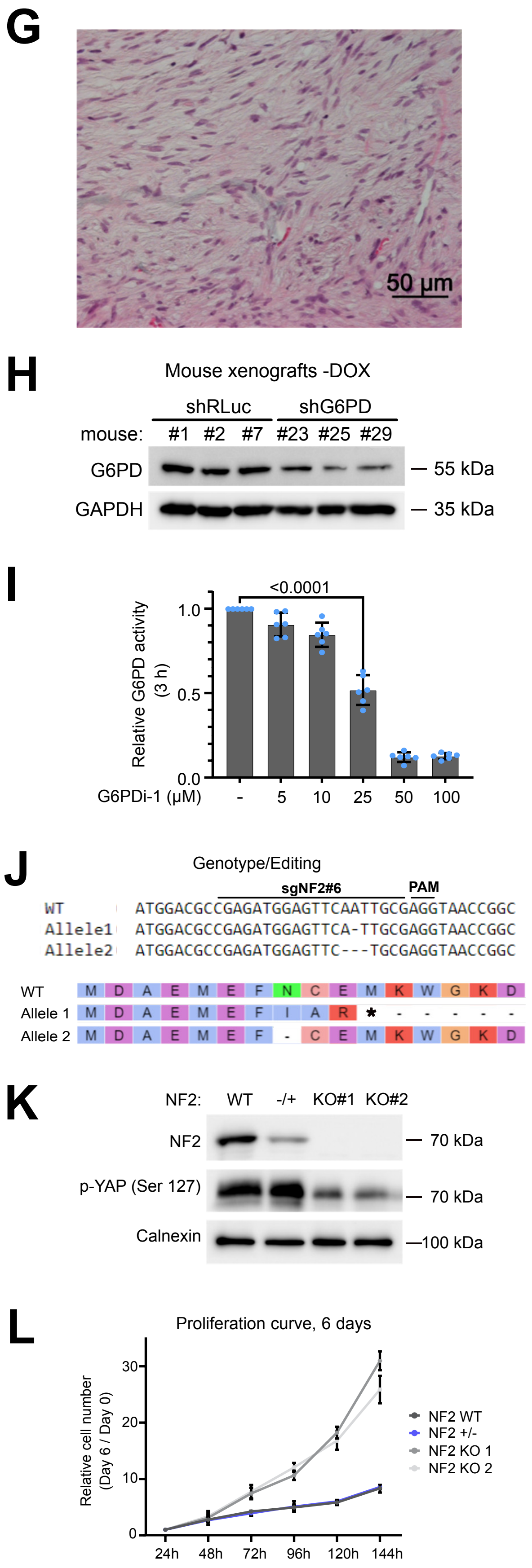

F

Yielded palpable tumors:	-Dox	+Dox
NF2KO#1 / shRLuc	10/10	10/10
NF2KO#1 / shG6PD#1	10/10	2/9
NF2KO#1 / shACSL3#1	9/10	10/10

Supplementary Figure 2: Support to main figure 2.

(A-C) siRNA-mediated knockdown of G6PD or ACSL3 causes synthetic lethality with NF2 loss-of-function in Schwann cells. (A) Relative cell number, assayed by CellTiter-Glo, normalized to control siRLuc. Dots = biological replicates. Bars = mean ± SD. Significance by two-way ANOVA and Tukey's multiple comparisons test. n=4 biological replicates. (B-C) Control immunoblots.

(D) Pharmacological inhibition of G6PD induces cell death of NF2-KO Schwann cells, assayed by flow cytometry as PI-positive cells 96hrs after treatment with G6PDi-1 (25μM). Bars = mean ± SD. Significance by one-way ANOVA and Dunnett's multiple comparisons test. n=3 biological replicates.

(E) Inhibition of G6PD causes synthetic lethality with NF2 loss-of-function also in Schwann cells growing in culture conditions with reduced growth factor stimulation (5% FBS). Ratio of cell number +G6PDi-1 inhibitor normalized to the -inhibitor condition, assayed by CellTiter Glo. Bars = mean ± SD. Significance by one-way ANOVA and Dunnett's multiple comparisons test. n=3 biological replicates.

(F) Tumor take rate for the indicated Schwann cell lines, either in the absence or presence of doxycycline in the drinking water to induce knockdown.

(G) High magnification histology image of the periphery of a G6PD-shRNA tumor - dox stained with hematoxylin and eosin.

(H) G6PD levels are reduced in mouse xenografts carrying the G6PD shRNA even without doxycycline. Shown are immunoblots on whole-tumor lysates from 3 randomly selected mice carrying xenografts of each genotype.

(I) A dose-response curve with G6PDi-1 reveals that 25µM G6PDi-1 causes roughly a 50% inhibition in G6PD activity. Dots = biological replicates. Bars = mean ± SD. Significance by unpaired t-test. n=6 biological replicates.

(J) Molecular characterization of the "NF2-/+" heterozygous Schwann cell line (DNA and resulting protein truncations). Allele 1 leads to a frameshift mutation and premature stop codon whereas allele 2 leads to loss of 1 amino acid.

(K) NF2-/+ cells have reduced NF2 protein levels but not reduced YAP phosphorylation, detected via immunoblotting with the indicated antibodies.

(L) NF2-/+ cells proliferate like NF2-wildtype cells, indicating that the remaining levels of NF2 in these cells are sufficient to provide full NF2 activity.

Source data are provided as a Source Data file.

Supplementary Figure 3

A

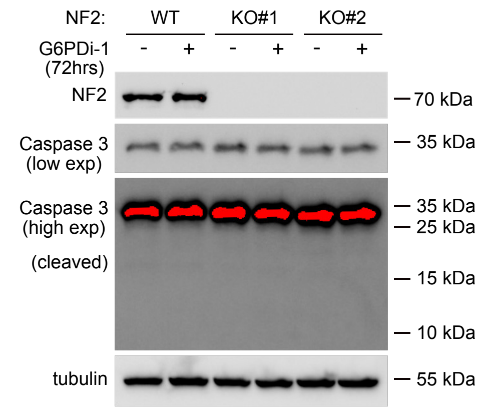

B

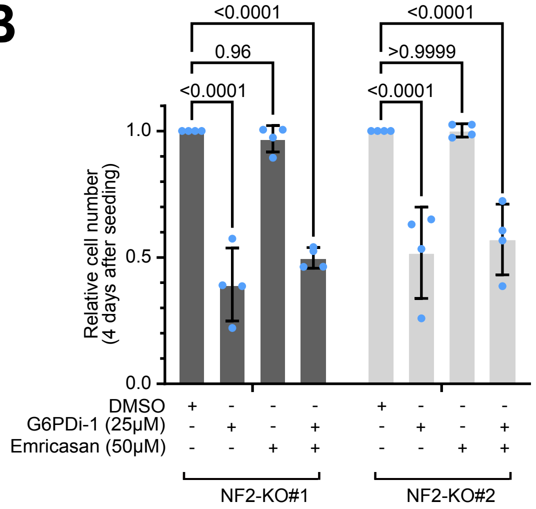

C

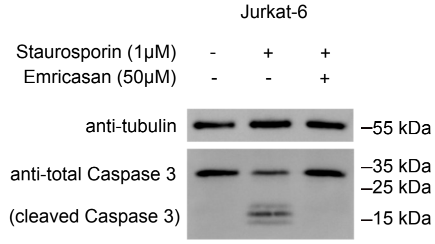

Supplementary Figure 3: NF2-KO cells die in response to G6PD inhibition via a caspase-independent cell death mechanism.

(A) NF2-KO cells treated with 25µM G6PDi-1, which induces cells death, does not lead to any visible caspase cleavage, detected by immunoblotting.

(B) Pan-caspase inhibitor Emricasan does not rescue reduced cell viability caused by G6PDi-1 in NF2-KO cells. Bars = mean ± SD. Significance by two-way ANOVA and Sidak's multiple comparisons test n=4 biological replicates.

(C) 25µM Emricasan efficiently blocks cleavage of caspase 3 induced by treatment with staurosporin.

Source data are provided as a Source Data file.

Supplementary Figure 4

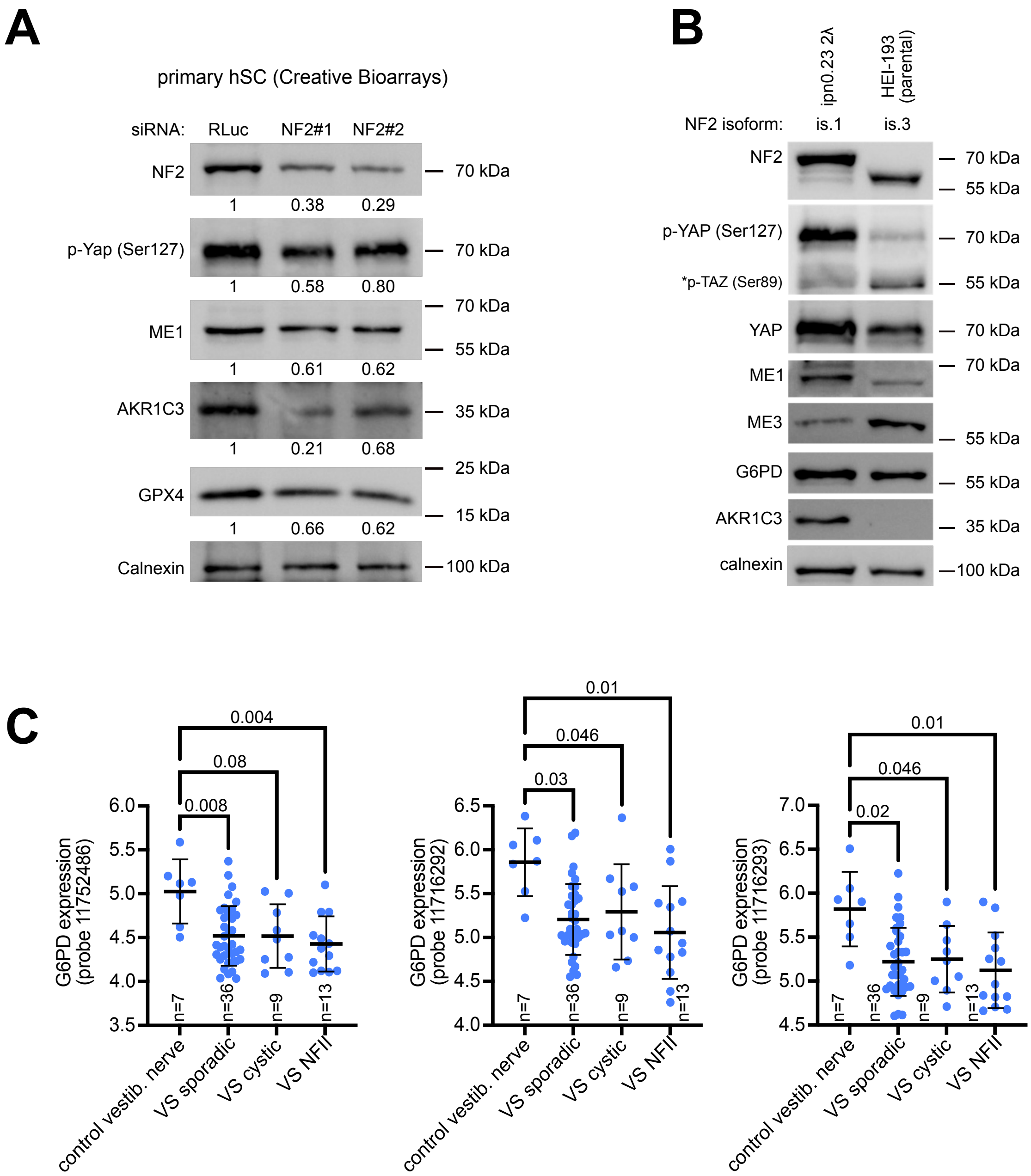

Supplementary Figure 4: Knockdown of NF2 reduces ME1 and AKR1C3 protein levels also in primary Schwann cells.

(A) Immunoblot analysis of indicated protein levels in primary Schwann cells 96hrs after siRNA-mediated knockdown of NF2 or RLuc (negative control). Numbers indicate protein levels normalized to calnexin.

(B) Compared to ipn ipn02.3 2λ cells, HEI-193 cells have expression of a hypomorphic "isoform 3" of NF2, reduced YAP phosphorylation, reduced ME1 levels and reduced AKR1C3 levels.

(C) G6PD levels are reduced in different types of vestibular schwannomas (VS: sporadic, cystic and NF2) compared to control vestibular nerve. Data are re-analyzed from [2]. Dots = individual patients. Bars = mean ± SD. Significance by Brown-Forsythe and Welch ANOVA and Dunnetts's multiple comparisons test. Source data are provided as a Source Data file.

Supplementary Figure 5

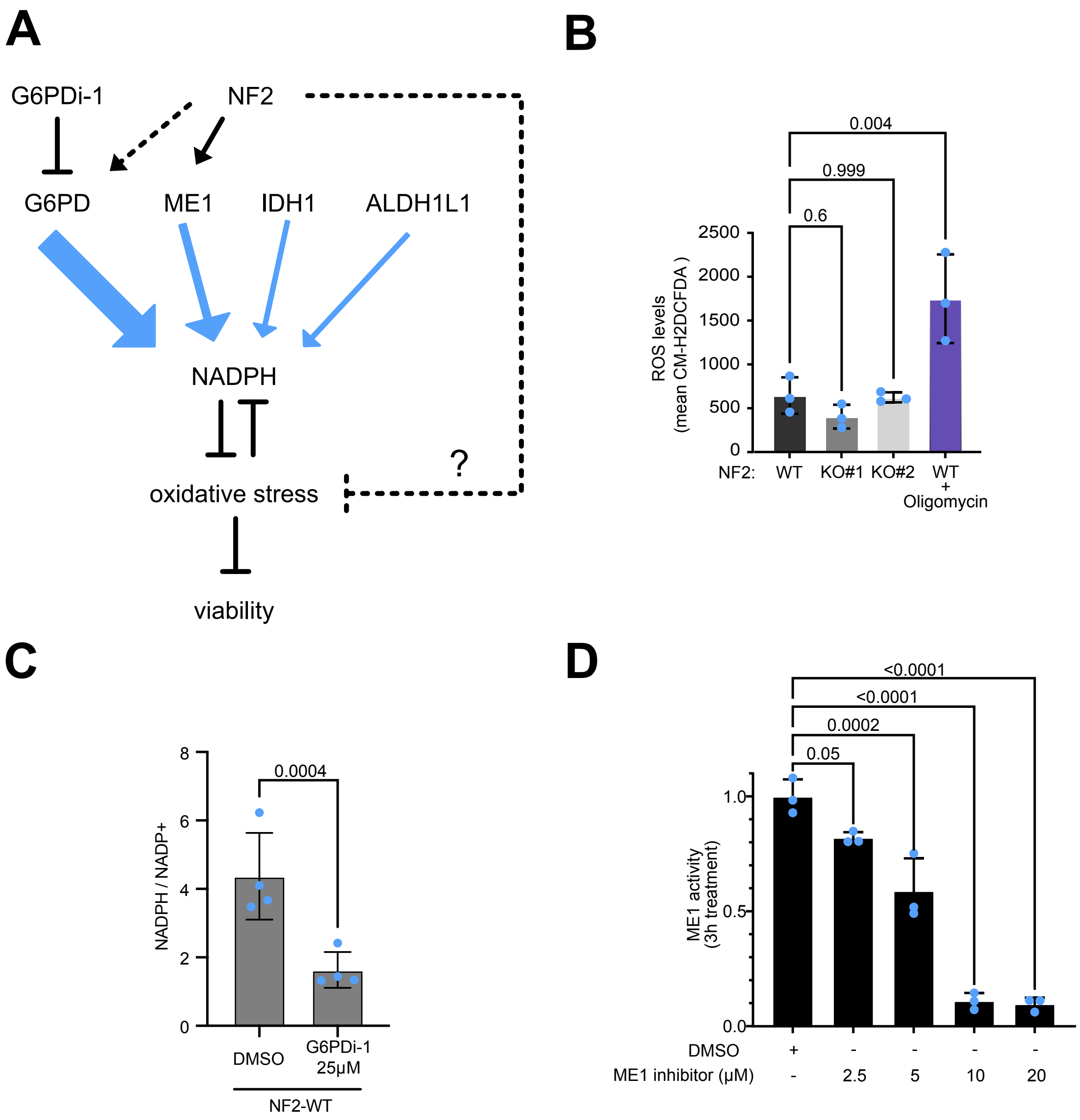

Supplementary Figure 5: Support to main figure 5.

(A) Mechanistic model for why inhibition of G6PD leads to lethality in NF2-KO cells. Loss of NF2 leads to a more oxidized cellular state in part due to reduced expression of NADPH-producing enzymes. As a result, NF2-KO cells rely more strongly on G6PD for NADPH production compared to NF2-WT cells. If G6PD is also inhibited pharmacologically in NF2-KO cells, then the cells die due to oxidative stress.

(B) NF2-KO Schwann cells do not have elevated basal ROS levels compared to isogenic NF2-WT cells. ROS levels assayed via flow cytometry using the ROS sensor CM-H2DCFDA (5µM-30min). Oligomycin (5µM-30min) was used as a positive control. Bars = mean ± SD. Significance by one-way ANOVA and Dunnett's multiple comparisons test. n=3 biological replicates

(C) Pharmacological inhibition of G6PD in NF2-WT Schwann cells leads to lower levels of reduced NADPH normalized to NADP+. n=4 biological replicates. Bars = mean ± SD. Significance by two-way ANOVA and Tukey's multiple comparisons test.

(D) Dose response curve with ME1 inhibitor identifies that 10µM ME1 inhibitor decreases ME1 enzymatic activity to the levels observed in NF2 KO cells (main Figure 4h). Results are normalized to enzymatic activity in wild type cells. Dots = biological replicates. Bars = mean ± SD. Significance by two-way ANOVA and Tukey's multiple comparisons test. n=3 biological replicates.

Source data are provided as a Source Data file.

Supplementary Figure 6

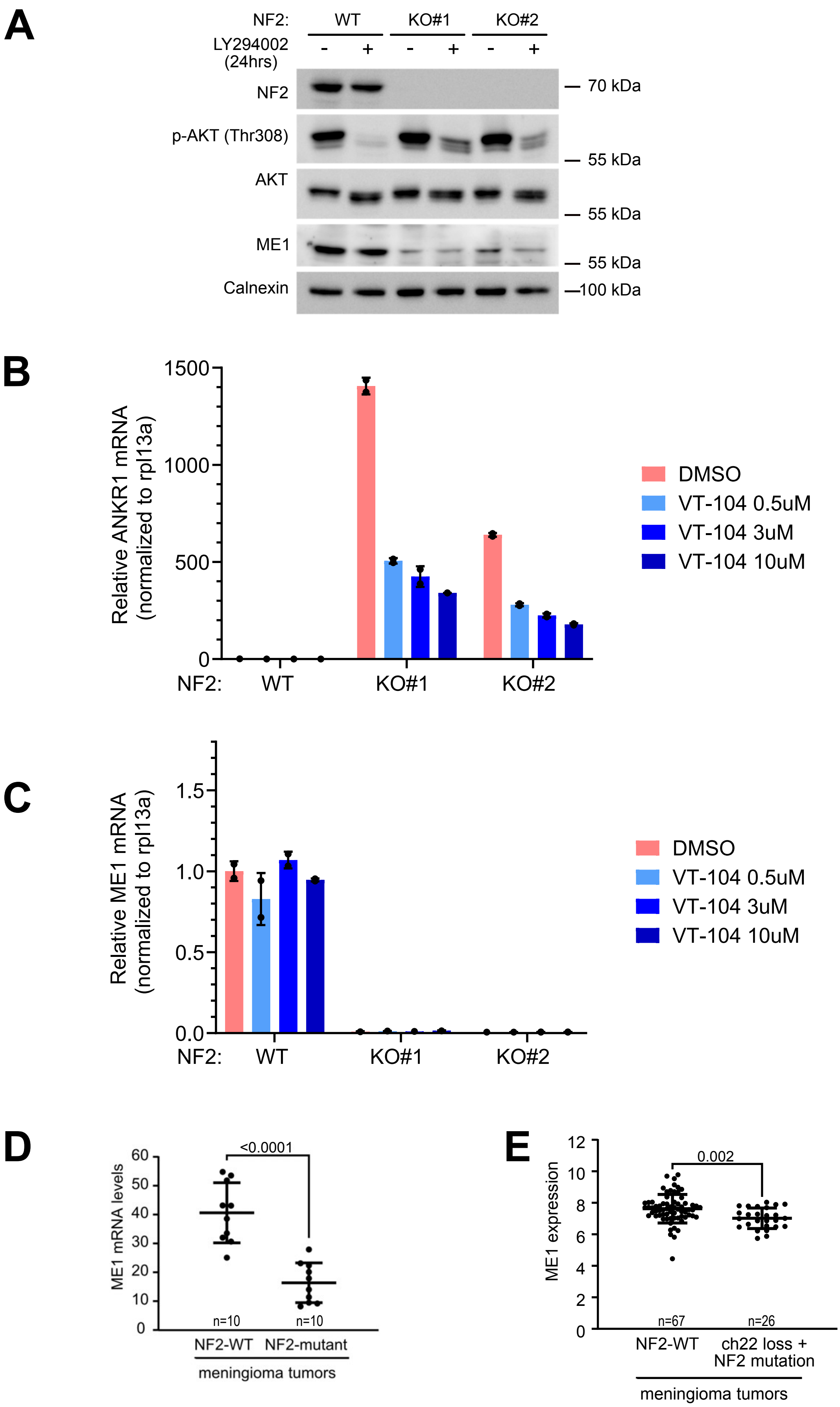

Supplementary Figure 6: Regulation of ME1 upon loss of NF2

(A) Inhibition of the PI3K pathway with LY294002 (30 μM, 24h) does not restore ME1 levels in the NF2-KO cells back to wildtype levels.

(B-C) Inhibition of TEAD transcription factors with VT-104 does not rescue the altered gene expression seen in NF2-KO cells back to NF2-WT levels. Cells were treated with the indicated concentrations of VT-104 for 6 h and then mRNA levels of ANKR1 (B) and ME1 (C) were measured by quantitative RT-PCR.

(D-E) ME1 mRNA levels are reduced in NF2 mutant meningiomas. (D) Re-analysis with GEO2R of data from [3]. Bars = mean ± SD. Significance by t-test. (E) Re-analysis with GEO2R of data from [4]. Significance by t-test.

Source data are provided as a Source Data file.

Supplementary Figure 7

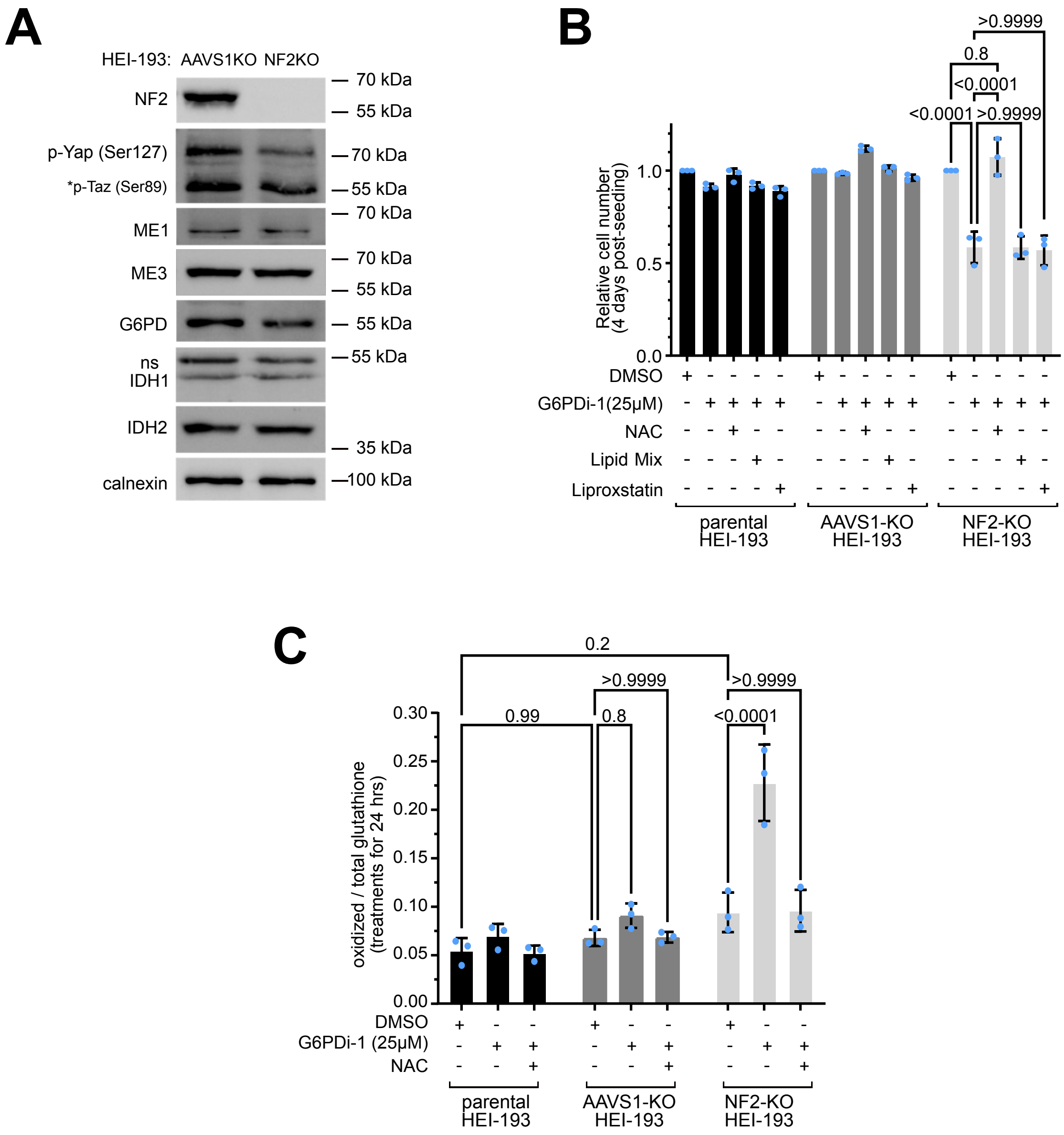

Supplementary Figure 7: Knockout of NF2 in HEI-193 cells causes them to die upon G6PD inhibition

(A) Knockout of NF2 in HEI-193 cells leads to reduced YAP phosphorylation but no further reduction in ME1 levels, which are already low (see Suppl. Fig. 4B).

(B) Pharmacological inhibition of G6PD with 25μM G6PDi-1 kills NF2-KO HEI-193 cells, and the lethality is rescued by NAC, indicating it is due to oxidative stress. Dots = biological replicates. Bars = mean ± SD. Significance by two-way ANOVA and Tukey's multiple comparisons test. n=3 biological replicates.

(C) G6PD inhibition leads to oxidized glutathione in NF2-KO HEI-193 cells, and this is rescued by treatment with NAC. Dots = biological replicates. Bars = mean ± SD. Significance by two-way ANOVA and Tukey's multiple comparisons test. n=3 biological replicates.

Source data are provided as a Source Data file.

Supplementary Figure 8

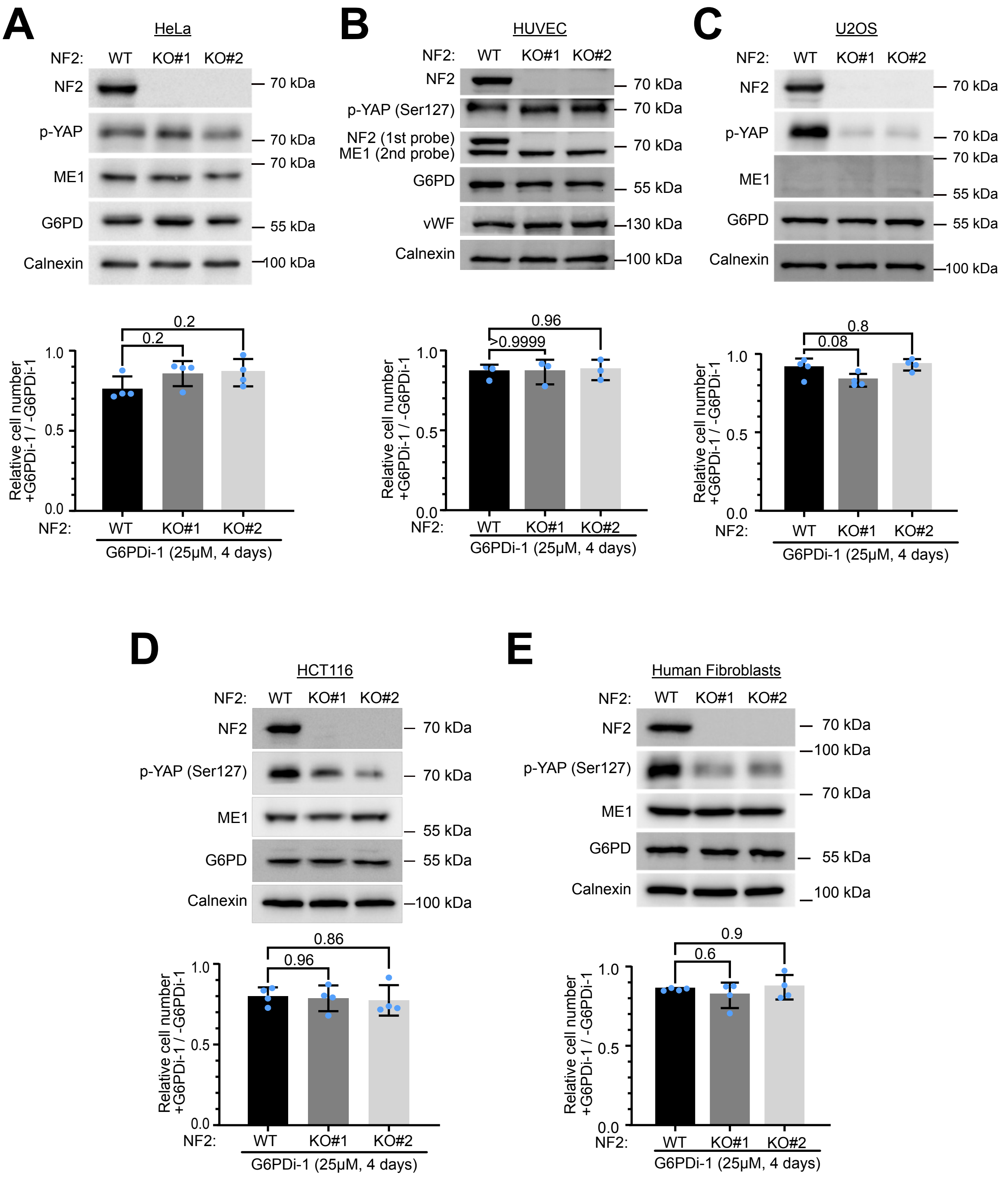

Supplementary Figure 8: Synthetic lethality between NF2 and G6PD is not observed in other cell lines tested.

NF2 was knocked-out in (A) HeLa cells, (B) immortalized human umbilical vein endothelial cells (HUVEC), (C) U2OS cells, (D) HCT116 cells and (E) immortalized human fibroblasts. Top panels show immuno-blots for the indicated proteins in each of the respective cell lines compared to the NF2-wildtype parental controls. Bottom panels show relative cell number quantified by CellTiter-Glo for cells treated + 25µM G6PDi-1 for 4 days, normalized cell treated without inhibitor for 4 days. Bars = mean ± SD. n=4 (panels A, C, D, E) or 3 (panel B) biological replicates. Statistical analysis shown is a one-way ANOVA and Dunnett’s multiple comparisons test.

Source data are provided as a Source Data file.

(Uncrropped Immunoblots continue on next page)

Uncropped Immunoblots

of Supplementary Figures

Suppl. Figure 1 Panel B

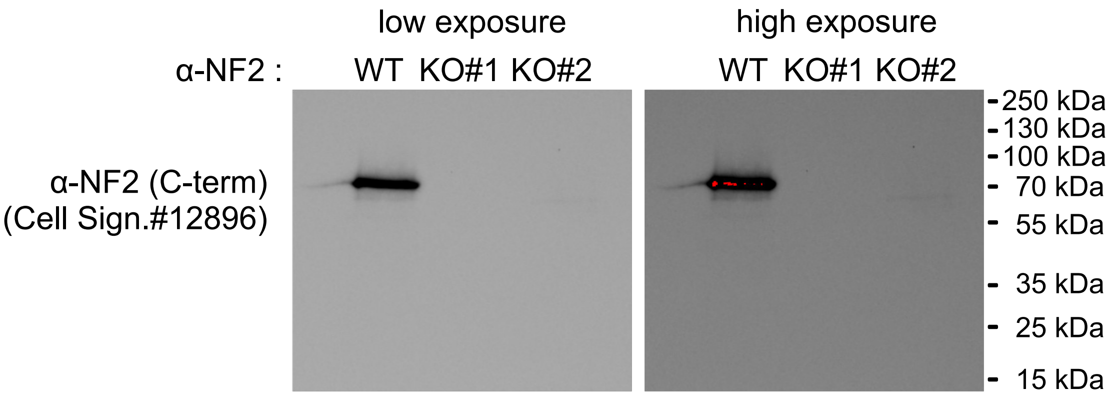

ED Figure 1 Panel E

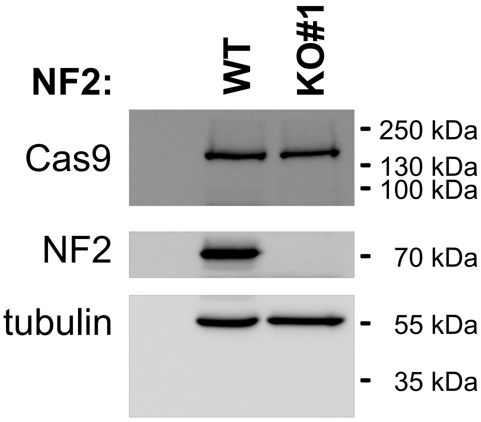

Suppl. Figure 2 Panel B

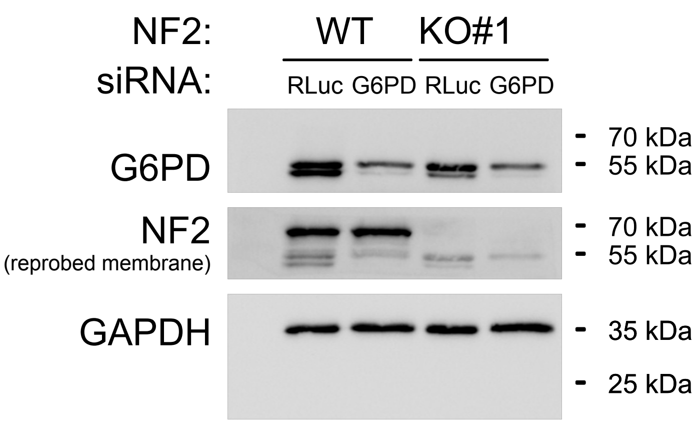

ED Figure 2 Panel C

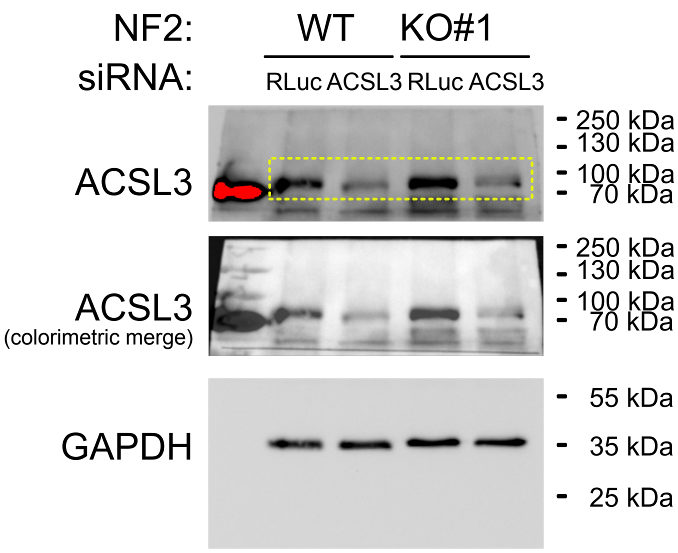

Suppl. Fig. 2H

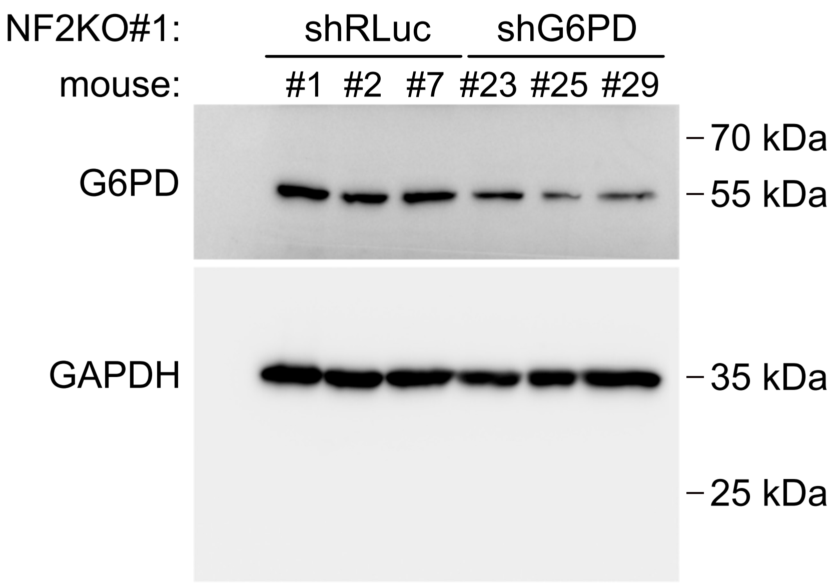

NF2KO#1:
shRLuc
shG6PD
mouse:
#1 #2 #7 #23 #25 #29
G6PD
GAPDH
70 kDa
55 kDa
35 kDa
25 kDa

Suppl. Figure 2 Panel K

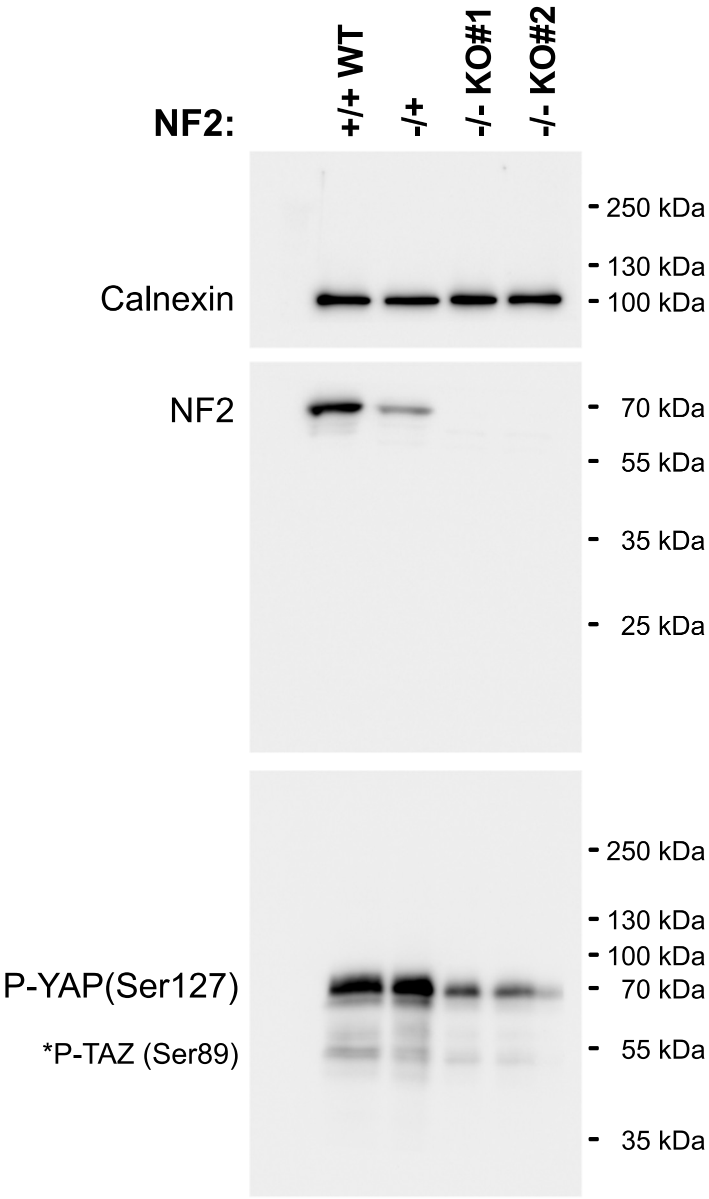

Suppl. Figure 3 Panel A

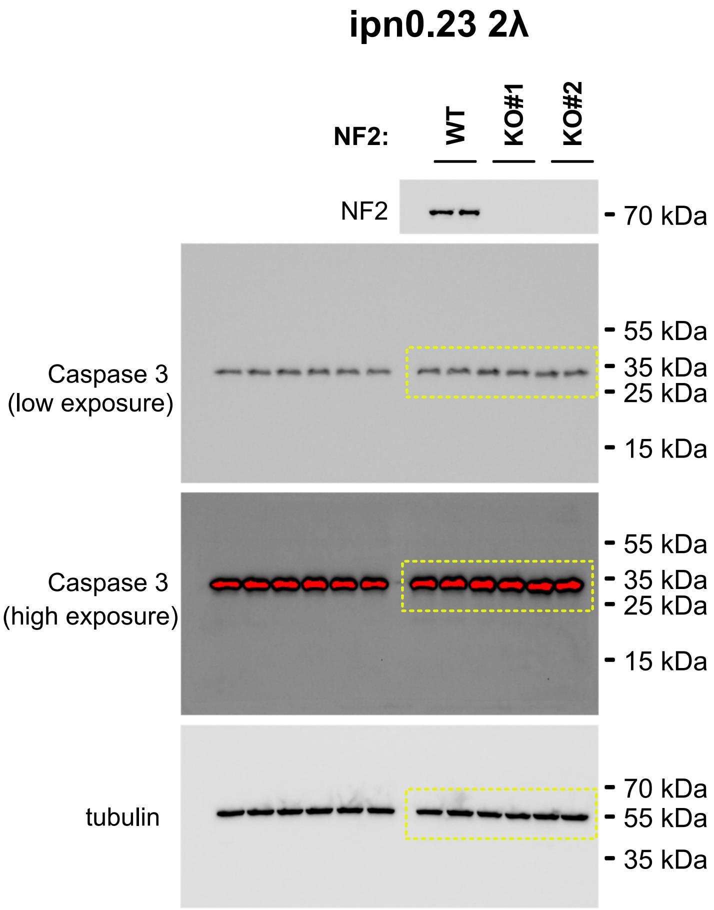

ED Figure 3 Panel C

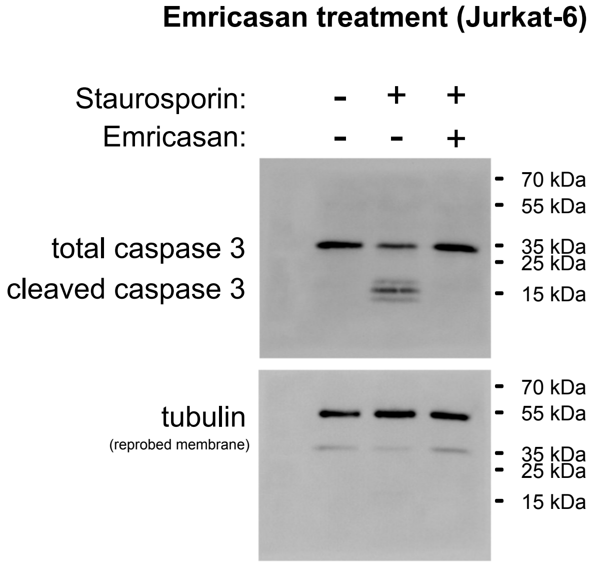

Suppl. Figure 4 Panel A

Creative Bioarrays:

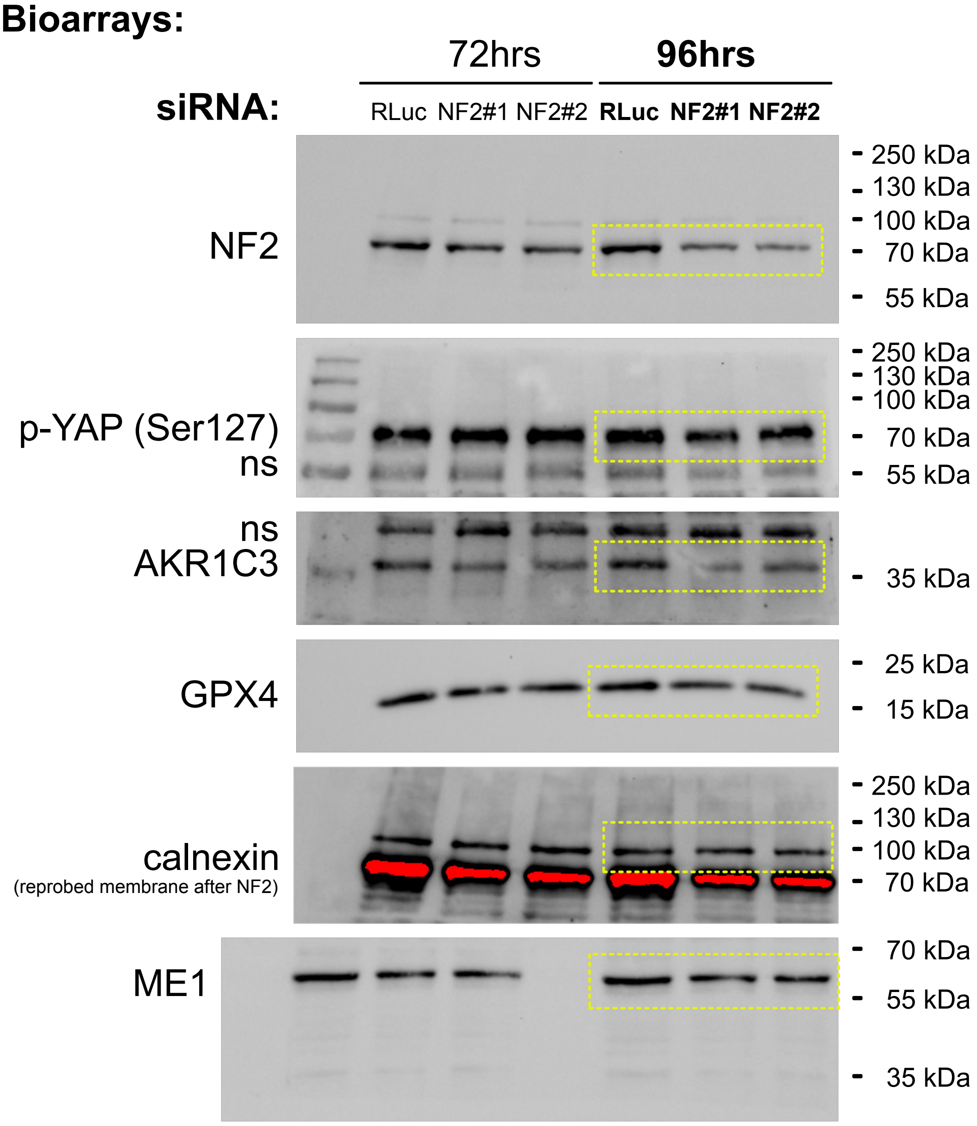

Suppl. Figure 4 Panel B

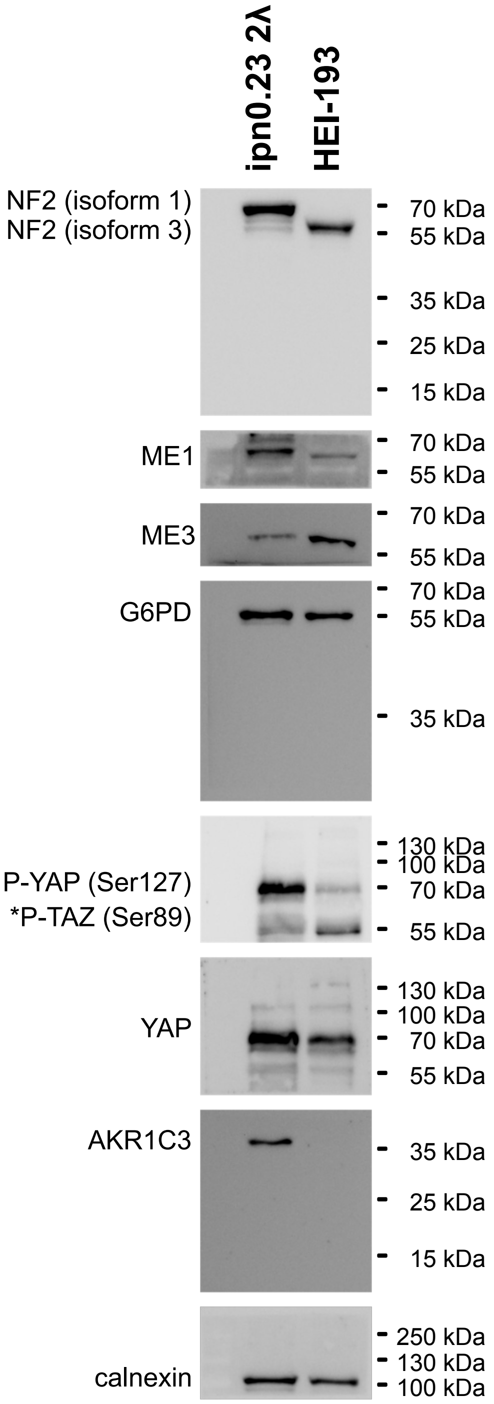

Suppl. Fig. 6A

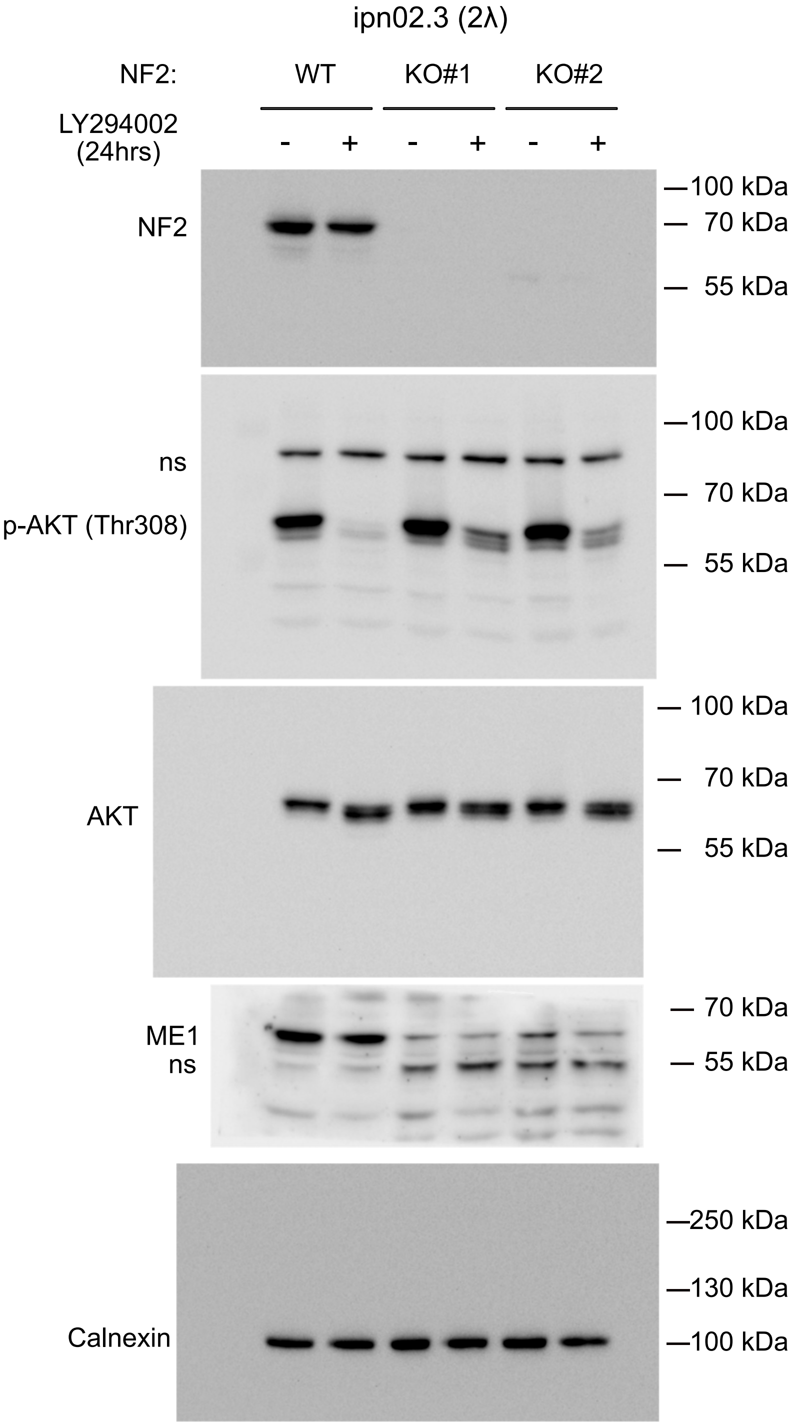

ipn02.3 (2λ)
NF2:
WT
KO#1
KO#2
LY294002
(24hrs)
- + - + - +
NF2
—100 kDa
— 70 kDa
— 55 kDa
ns
p-AKT (Thr308)
—100 kDa
— 70 kDa
— 55 kDa
AKT
— 100 kDa
— 70 kDa
— 55 kDa
ME1
ns
— 70 kDa
— 55 kDa
Calnexin
—250 kDa
—130 kDa
—100 kDa

Suppl. Figure 7 Panel A

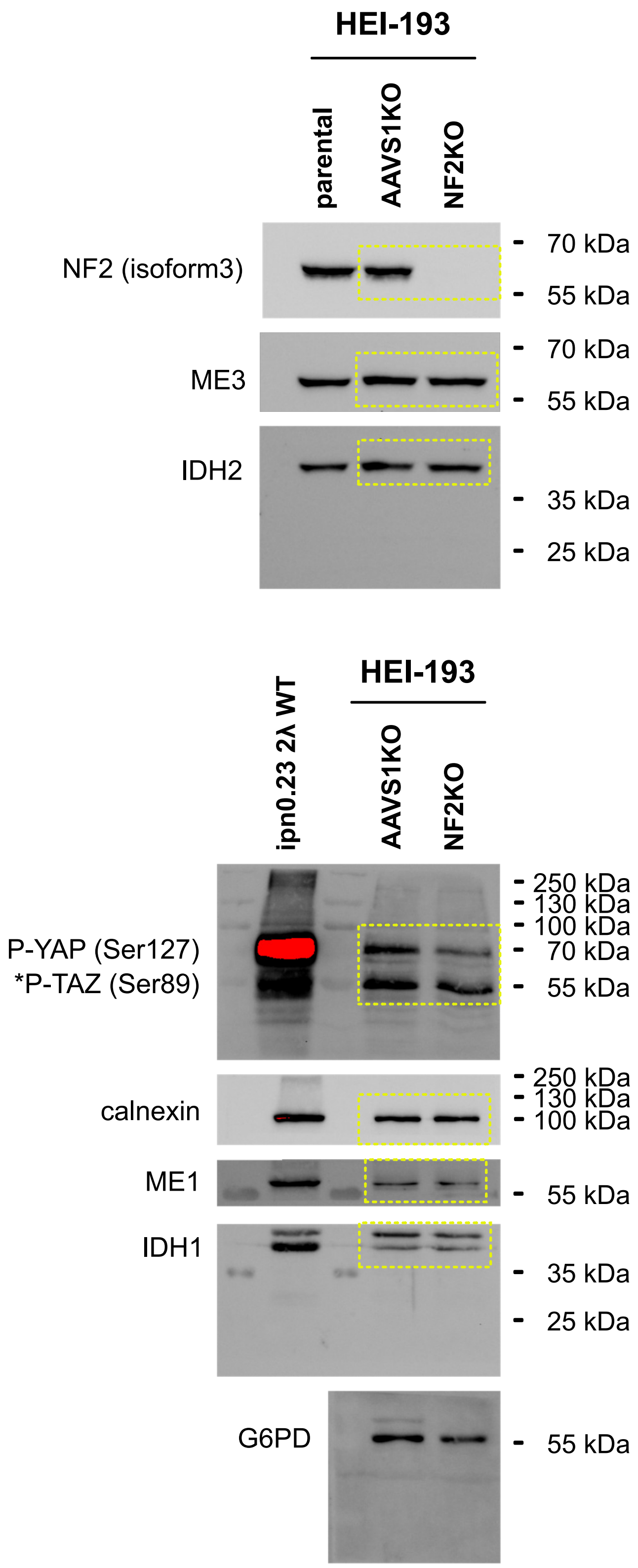

Suppl. Figure 8 Panel A

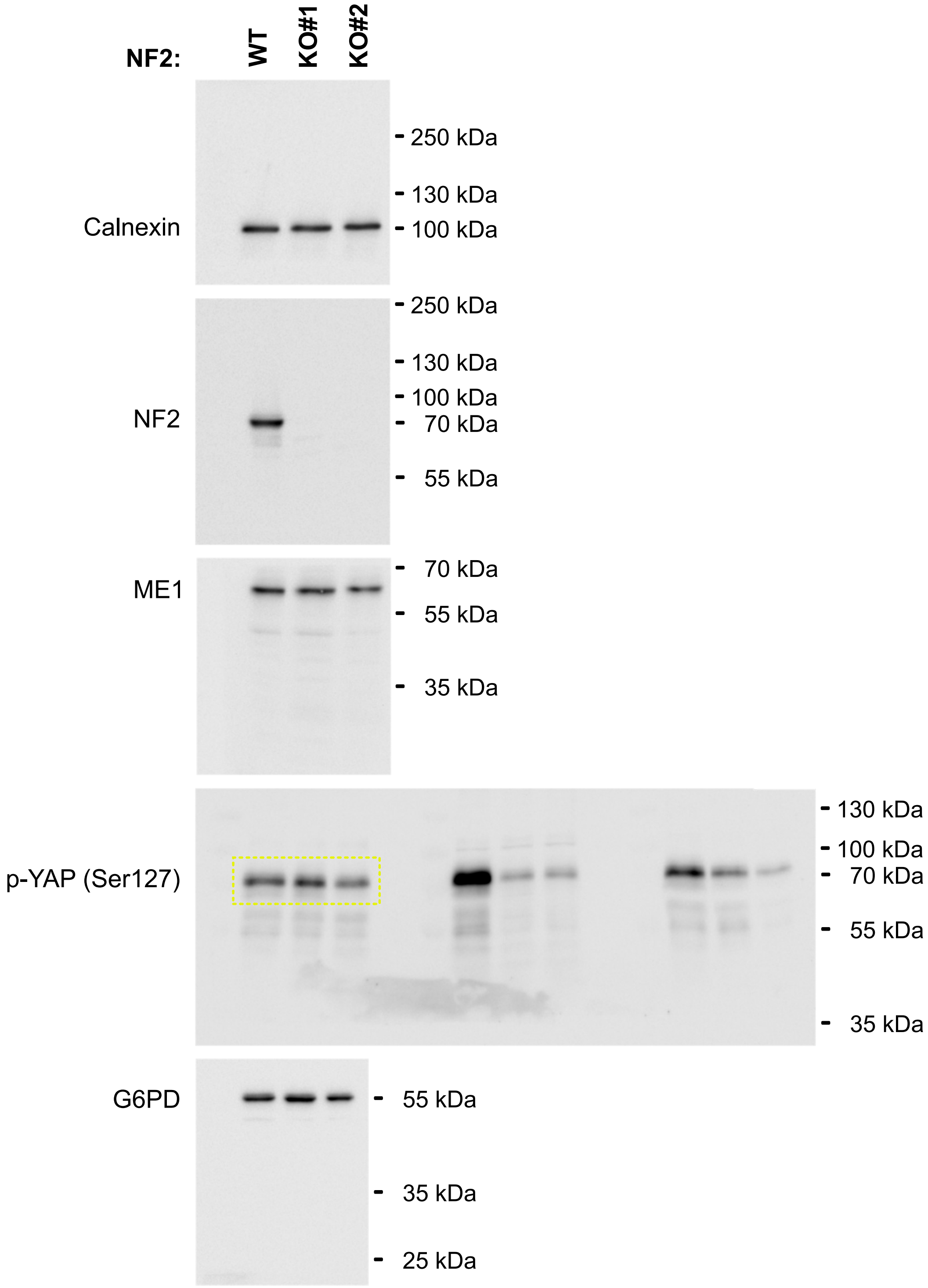

Suppl. Figure 8 Panel B

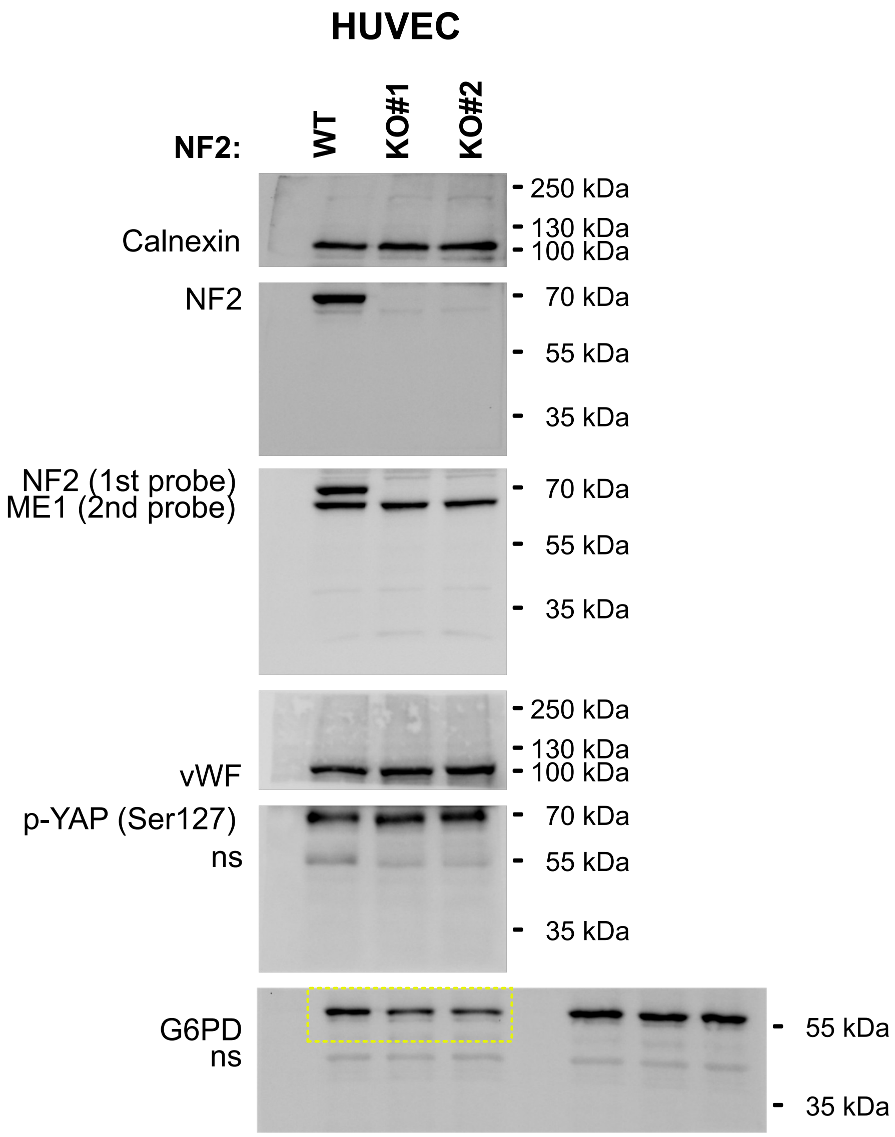

Suppl. Figure 8 Panel C

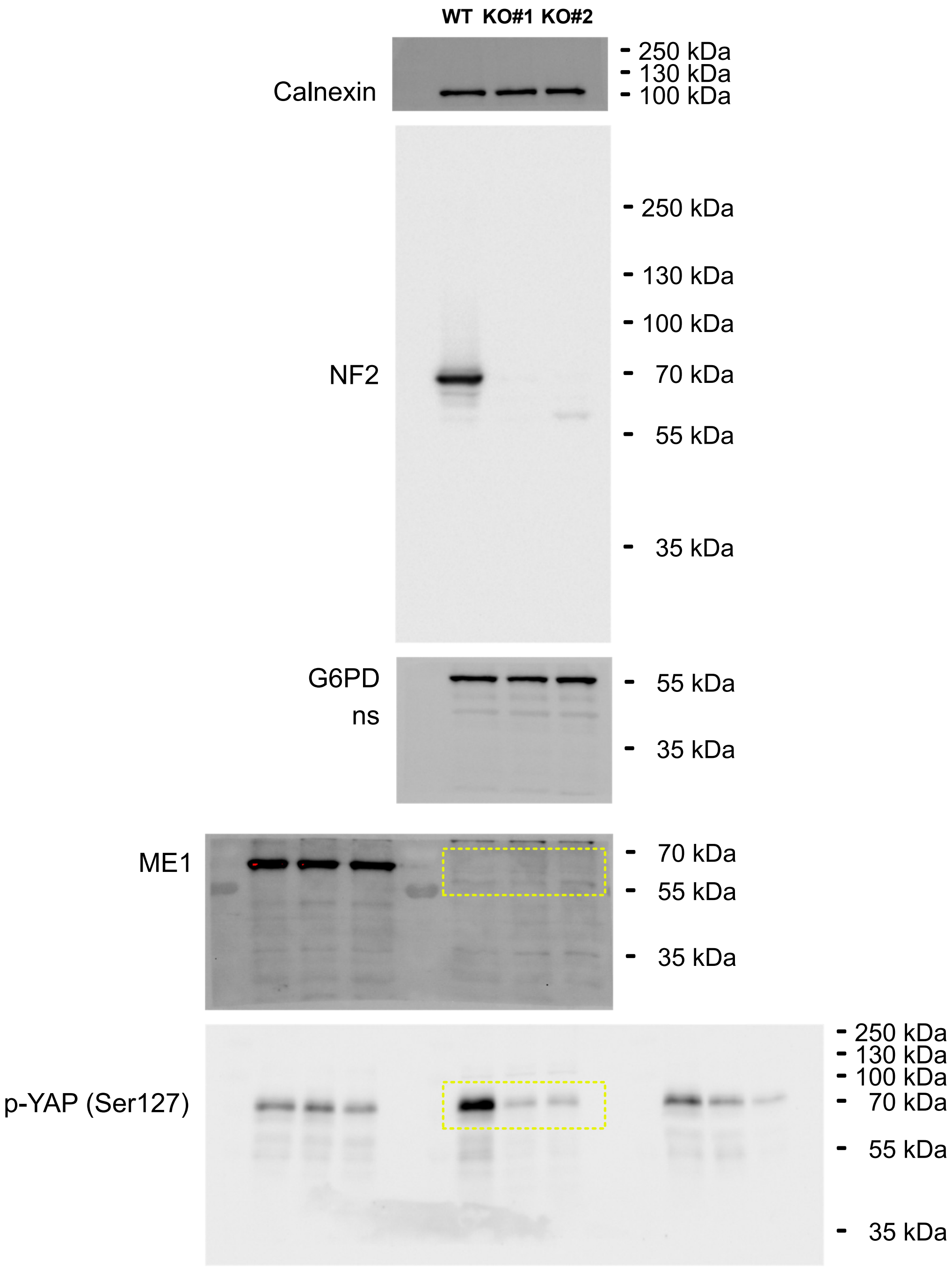

Suppl. Figure 8 Panel D

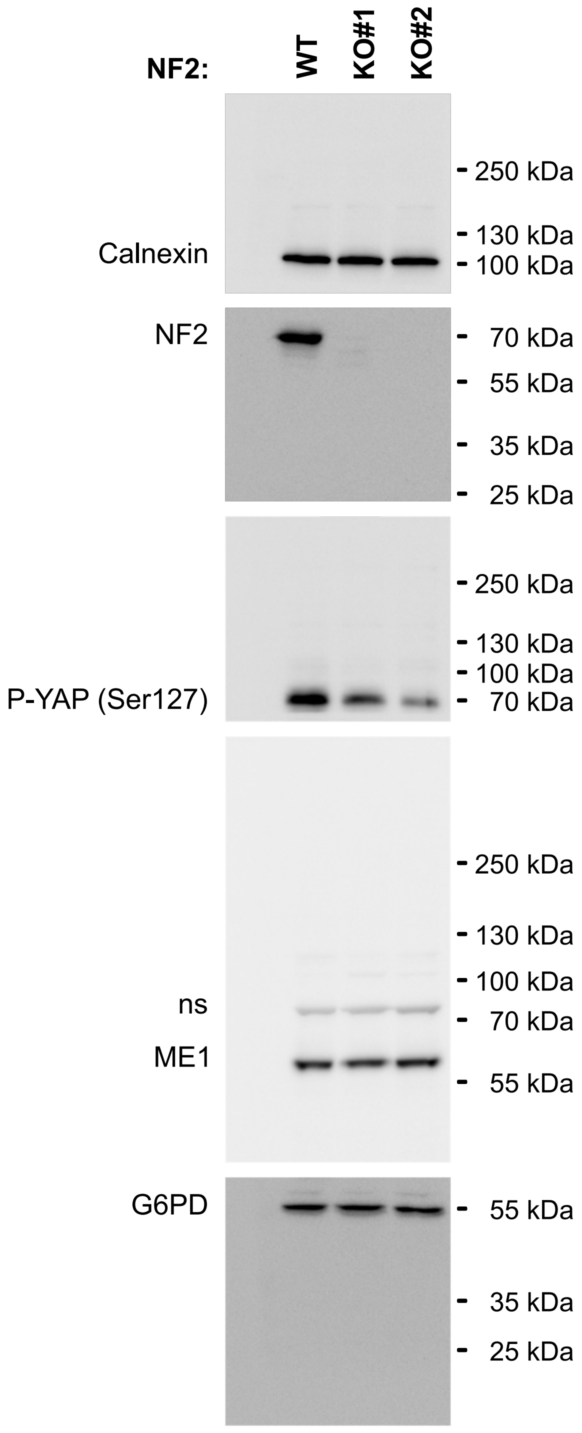

Suppl. Figure 8 Panel E

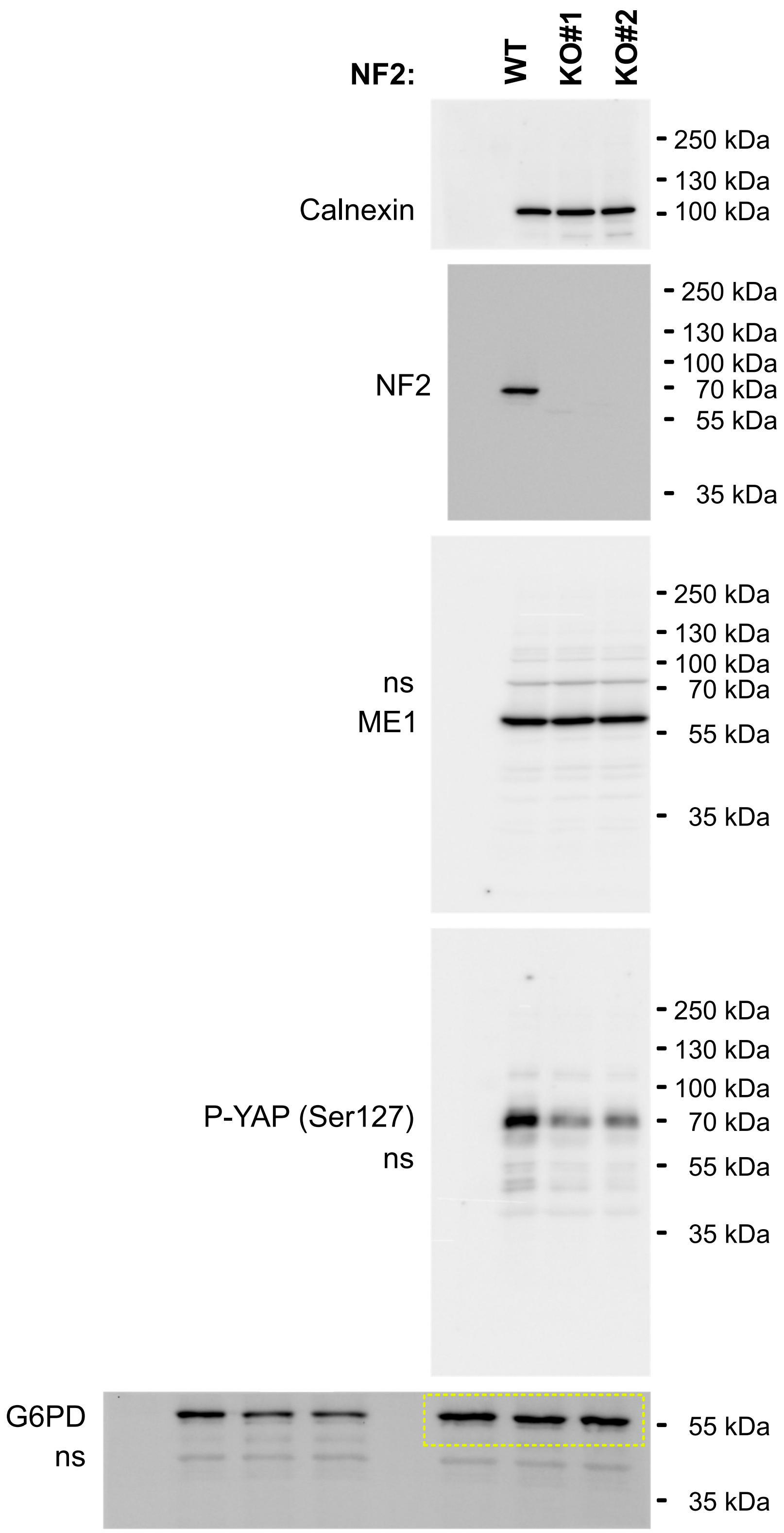

Supplementary Information References

1. Ge SX, Jung D, Yao R. ShinyGO: a graphical gene-set enrichment tool for animals and plants. *Bioinformatics* **36**, 2628-2629 (2020).

2. Gugel I*, et al.* Contribution of mTOR and PTEN to Radioresistance in Sporadic and NF2-Associated Vestibular Schwannomas: A Microarray and Pathway Analysis. *Cancers* **12**, (2020).

3. Tsitsikov EN*, et al.* Specific gene expression signatures of low grade meningiomas. *Frontiers in oncology* **13**, 1126550 (2023).

4. Clark VE*, et al.* Genomic analysis of non-NF2 meningiomas reveals mutations in TRAF7, KLF4, AKT1, and SMO. *Science* **339**, 1077-1080 (2013).
